# Plasmid-Encoded Transferable *mecB*-Mediated Methicillin Resistance in *Staphylococcus aureus*

**DOI:** 10.3201/eid2402.171074

**Published:** 2018-02

**Authors:** Karsten Becker, Sarah van Alen, Evgeny A. Idelevich, Nina Schleimer, Jochen Seggewiß, Alexander Mellmann, Ursula Kaspar, Georg Peters

**Affiliations:** University Hospital Münster, Münster, Germany (K. Becker, S. van Alen, E.A. Idelevich, N. Schleimer, J. Seggewiß, A. Mellmann, U. Kaspar, G. Peters);; Cells in Motion, Münster (G. Peters)

**Keywords:** Staphylococcaceae, methicillin-resistant Staphylococcus aureus, Macrococcus, microbial genome, plasmids, antibacterial agents, Staphylococcus aureus, bacteria, staphylococci, MRSA, mecB, antimicrobial resistance

## Abstract

During cefoxitin-based nasal screening, phenotypically categorized methicillin-resistant *Staphylococcus aureus* (MRSA) was isolated and tested negative for the presence of the *mecA* and *mecC* genes as well as for the SCC*mec*-*orfX* junction region. The isolate was found to carry a *mecB* gene previously described for *Macrococcus caseolyticus* but not for staphylococcal species. The gene is flanked by β-lactam regulatory genes similar to *mecR*, *mecI*, and *blaZ* and is part of an 84.6-kb multidrug-resistance plasmid that harbors genes encoding additional resistances to aminoglycosides (*aacA-aphD*, *aphA*, and *aadK*) as well as macrolides (*ermB*) and tetracyclines (*tetS*). This further plasmidborne β-lactam resistance mechanism harbors the putative risk of acceleration or reacceleration of MRSA spread, resulting in broad ineffectiveness of β-lactams as a main therapeutic application against staphylococcal infections.

Staphylococcal cassette chromosome *mec* (SCC*mec*)–mediated β-lactam resistance resulting from production of an additional penicillin-binding protein (PBP) 2a drastically limits the treatment options in cases of hospital- and community-related infections by staphylococci, leading to increased illness, death, and socioeconomic costs (*1*,*2*). Besides methicillin-resistant coagulase-negative staphylococci, notorious for foreign body–associated infections, methicillin-resistant *Staphylococcus aureus* (MRSA) strains are a global public health priority, despite some countries in Europe reporting stabilizing or decreasing MRSA rates (*3*–*5*). Since the initial reports of MRSA in 1961, several epidemic waves have resulted in threats of healthcare-, community-, and livestock-associated MRSA (*6*–*9*).

For staphylococci, 2 PBP 2a-encoding genes, *mecA* and *mecC*, including several allotypes, have been described as chromosomally located genetic bases for phenotypic methicillin resistance (*10*–*14*). In contrast, *mecB*, originally described as *mecA_m_*, was reported as part of a probable primordial form of a methicillin resistance gene complex often found in a transposon *mec* complex (Tn6045) in *Macrococcus caseolyticus*, a colonizer of animal skin (*15*,*16*). Just recently, a *mecD* gene, most closely related to *mecB*, has been detected in bovine and canine *M. caseolyticus* isolates (*17*).

The impact of plasmidborne resistance for staphylococci is abundantly demonstrated for β-lactamase–mediated penicillin resistance. Resistance rates are >60% in human *S. aureus* isolates from the general population and >90% from hospital-related cases, regardless of the clinical background (*18*,*19*). In contrast to frequent interstrain and interspecies transmission of resistance plasmids by conjugation or transduction, only a relatively low rate of spontaneous horizontal transfer of SCC*mec* elements is assumed, resulting in still-manageable and controllable MRSA rates if prevention measures are adequate (*20*–*24*). However, transferable methicillin resistance might bear the consequence of an almost complete loss of β-lactam drugs as the most efficient class of antibacterial drugs for treatment of staphylococcal infections. Here, we report both a plasmid-encoded, and thereby transferable, methicillin resistance encoded by *mecB* and the occurrence of this gene in an isolate of the genus *Staphylococcus*.

## Methods

### Strain Detection and Identification

At the University Hospital of Münster, Germany, MRSA is generally cultured, identified, and differentiated by routine microbiological diagnostic methods using dextrose broth enrichment; chromID MRSA selective agar (bioMérieux, Marcy-l’Étoile, France), which contains cefoxitin; VITEK 2 automated system (bioMérieux) applying the antimicrobial susceptibility test card AST-P632; PBP2a detection kit (PBP2a Culture Colony Test, Alere, San Diego, CA, USA); *S. aureus*–specific PCR targeting *mecA*/*mecC* (GenoType MRSA, Hain-Lifescience, Nehren, Germany); and matrix-assisted laser desorption/ionization time-of-flight mass spectrometry (Microflex-LT system, MALDI-Biotyper 3.0; Bruker Daltonik, Bremen, Germany). In February 2016, an *S. aureus* isolate (which we numbered UKM4229) was recovered during routine MRSA screening. The isolate displayed a β-lactam–resistant phenotype without carrying the methicillin resistance genes *mecA* or *mecC*. For further characterizations, isolate UKM4229 was stored at −80°C and was cultivated on chromID MRSA agar (bioMérieux) at 37°C.

### Genetic Analysis

We extracted genomic DNA from *S. aureus* isolate UKM4229 using the QIAamp DNA Mini Kit (QIAGEN, Hilden, Germany) according to the manufacturer’s instructions. We isolated plasmid DNA with the PrepEase MiniSpin Plasmid Kit (Affymetrix USB, Santa Clara, CA, USA) following the protocol standards. For both plasmid and genomic DNA, we applied lysostaphin (20 µg/mL) (Wakchemie, Steinbach, Germany) for bacterial cell lysis. We performed multilocus sequence typing and *spa* gene typing initially as described elsewhere (*25*,*26*) and confirmed our results later by analysis of whole-genome sequencing (WGS) and DNA microarray data (discussed later in this article). We analyzed DNA sequences using RidomStaphType and SeqSphere+ (Ridom GmbH, Münster, Germany). Applying DNA microarray analysis (IdentiBAC Microarray; Alere Technologies GmbH, Jena, Germany), we identified resistance and virulence determinants and checked genotyping results.

### Molecular Confirmation of Methicillin Resistance

Using PCR, we tested for the presence of methicillin resistance genes *mecA* and *mecC* (*27*,*28*) as well as *mecB*. DNA sequences of PCR oligonucleotides are given in Table 1. Oligonucleotides for *mecB* were made on basis of the plasmid pMCCL2 of *M. caseolyticus* (GenBank accession no. NC_011996.1). We performed PCR reactions using the following protocol for *mecA*: 5 min at 95°C; 40 cycles of 0.5 min at 95°C, 0.5 min at 55.5°C, and 0.75 min at 72°C; and final elongation of 7 min at 72°C. The protocol for *mecB* was 5 min at 95°C; 35 cycles of 0.5 min at 95°C, 0.5 min at 57°C, and 2.5 min at 72°C; and final elongation of 7 min at 72°C. The protocol for *mecC*: 5 min at 95°C; 40 cycles of 0.5 min at 95°C, 0.5 min at 59.3°C, and 2 min at 72°C; and final elongation of 7 min at 72°C.

**Table 1:** Oligonucleotides used in study of methicillin resistance genes in *Staphylococcus aureus*

Gene	Oligonucleotide	Nucleotide sequence, 5′ → 3′	Melting temperature	Source
*mecA*	*mec*5	AAAATCGATGGTAAAGGTTGGC	55.5°C	(*29*)
	*mec*6	AGTTCTGCAGTACCGGATTTGC		
*mecC*	*mec*AL3	TCAAATTGAGTTTTTCCATTATCA	59.3°C	This study
	*mec*AL4	AACTTGGTTATTCAAAGATGACGA		
*mecB*	*mecB*-for	TTAACATATACACCCGCTTG	57°C	This study
	*mecB*-rev	TAAAGTTCATTAGGCACCTCC		

### Antibiotic Drug Susceptibility Testing

We determined the MIC of cefoxitin for *S. aureus* isolate UKM4229 by the reference broth microdilution method according to the International Organization for Standardization (ISO) 20776-1 guideline (https://www.iso.org/standard/41630.html), as required by the European Committee on Antimicrobial Susceptibility Testing (EUCAST) and the Clinical and Laboratory Standards Institute (CLSI). Cefoxitin (Sigma Aldrich, Taufkirchen, Germany) was tested in 2-fold concentrations (0.25–128 µg/mL). We subcultured the isolate and incubated it overnight before testing.

We investigated the susceptibility profile of UKM4229 by determining MICs of various β-lactam and non–β-lactam antibiotic drugs (Table 2) using the gradient diffusion method (Etest; bioMérieux) according to the manufacturer’s instructions. As recommended, the inoculated plates were incubated at 35°C for 18 ± 2 hours. In addition, we tested oxacillin using conditions for increased expression of methicillin resistance, as reported for *mecA* isolates (*30*): Mueller-Hinton agar supplemented with 2% saline, incubation at 30°C, and prolonged incubation up to 48 h. We investigated the applicability of a commercial automated susceptibility testing device to recognize methicillin resistance due to presence of *mecB* in *S. aureus* by using the VITEK 2 system. We evaluated the in vitro activity of the endolysin HY-133 against UKM4229 using the broth microdilution method in accordance with ISO 20776-1 guidance (https://www.iso.org/standard/41630.html), as described elsewhere (*31*,*32*). In brief, we tested 2-fold final concentrations of HY-133 ranging from 0.06 µg/mL to 8 µg/mL using 1–5 × 10^5^ CFU/mL suspension of UKM4229 in cation-adjusted Mueller-Hinton broth. The MICs were read after incubation at 35°C for 18 ± 2 h.

**Table 2:** Susceptibility to antimicrobial drugs of *Staphylococcus aureus* isolate UKM4229 from a 67-year-old cardiology inpatient who had no signs of infection, Münster, Germany*

Antimicrobial class and agent	Median MIC, µg/mL	Category
β-lactams		
Penicillins		
Benzylpenicillin	1.5	R
Ampicillin	3	
Ampicillin/sulbactam	2	
Piperacillin	6	
Piperacillin/tazobactam	3	
Oxacillin	12	
Oxacillin†	4/4	
Cephalosporins		
Cefoxitin	32	R
Cephalothin	2	
Cefuroxime	3	
Ceftriaxone	24	
Cefepime	6	
Ceftobiprole	2	S
Ceftaroline	0.5	S
Carbapenems		
Imipenem	0.032	
Non–β-lactams		
Glycopeptides		
Vancomycin	1	S
Lipoglycopeptides		
Telavancin	0.012	
Lipopeptides		
Daptomycin	0.19	S
Fluoroquinolones		
Levofloxacin	0.19	S
Macrolides		
Erythromycin	>256	R
Lincosamids		
Clindamycin	>256	R
Oxazolidiones		
Linezolid	1	S
Rifamycins		
Rifampin	0.008	S
Phosphonic acid derivatives		
Fosfomycin	<0.064	S
Streptogramins		
Quinupristin/dalfopristin	0.5	S
Tetracyclines		
Tetracycline	12	R
Glycylcyclines		
Tigecycline	0.125	S
Folate pathway inhibitors		
Trimethoprim/sulfamethoxazole	0.047	S
Aminoglycosides		
Gentamicin	24	R
Pseudomonic acids		
Mupirocin	0.19	S
Fusidanes		
Fusidic acid	0.094	S
Bacteriophage endolysins		
HY-133	0.25	

*Tested by using the gradient diffusion method. S, susceptible; R, resistant (according to EUCAST [www.eucast.org] for antibiotic drugs with available breakpoints) †Conditions: 2% NaCl, 30°C; 18 h/48 h. MIC at regular reading after 18 ± 2 h/MIC after 48 h.

We performed all experiments in triplicate on different days and calculated the median MIC values. We used *S. aureus* ATCC 29213 as a quality control strain on every testing day. For the antibiotic drugs we used, the MICs for the quality control strain were within acceptable limits throughout the testing.

### Whole-Genome Sequencing

For the PacBio RS II platform (Pacific Biosciences, Menlo Park, CA, USA), we extracted staphylococcal DNA using the Genomic-tip 20/G Kit (QIAGEN) according to the manufacturer’s instructions, excep that we applied lysostaphin (20 µg/mL) (Wakchemie) for bacterial cell lysis. We sequenced the extracted high-quality, double-stranded DNA (5 µg) using P6-C4 chemistry on the PacBio RS II instrumentation using 4-hour movie collection and 110 pmol/L of complexed 20-kb SMRTbell library. We performed the initial de novo assembly using the HGAP3 v2.3.0 Assembler (Icahn Institute for Genomics and Multiscale Biology, Icahn School of Medicine at Mount Sinai, New York, NY, USA). We annotated the assembled genome through the GenDB pipeline (*33*). We verified questionable sequences within the plasmids by applying PCR (LA-Taq-DNA-Polymerase; Takara, Frankfurt am Main, Germany) and Sanger sequencing (Eurofins Genomics, Ebersberg, Germany).

## Results

During routine MRSA screening, we recovered an *S. aureus* isolate UKM4229 from a combined nasal-throat swab of a 67-year-old male cardiology inpatient who had no signs of infection. We isolated colonies with typical appearance for presumptive MRSA from a chromogenic MRSA selective agar and identified them as *S. aureus* by VITEK 2, matrix-assisted laser desorption/ionization time-of-flight mass spectrometry, and PCR. Discrepancies between phenotypic detection of methicillin resistance by VITEK 2 and negative results of a PBP2a detection kit, as well as negative *mecA* and *mecC* test results, by commercial and in-house PCRs led to the detection of a *mecB*-encoded methicillin resistance.

*S. aureus* isolate UKM4229 showed a median MIC of 32 µg/mL for cefoxitin, as determined by broth microdilution and gradient diffusion tests. The MICs of other antibiotics, as well as correspondent interpretative categories, are shown in Table 2; the resistance gene profile is given in Technical Appendix Table 1. Optimal oxacillin testing conditions previously reported to increase expression of methicillin resistance in *mecA* isolates (*30*) unexpectedly led to lower oxacillin MIC values for UKM4229 (Table 2). A novel anti–*S. aureus* agent in development, the recombinant phage endolysin HY-133 (Hyglos, Bernried, Germany) (*31**,**32*), was also active. VITEK 2 recognized *mecB*-associated methicillin resistance by oxacillin MIC determination and cefoxitin screening.

Although *mecA*/*mecC* PCR did not yield amplicons, *mecB*-specific PCR applying total and plasmid DNA resulted in a PCR product similar to those of *M. caseolyticus* isolate AM20CR01 (from a clam; isolate provided from J.E. Rubin, Institute of Veterinary Microbiology, University of Saskatchewan, Saskatchewan, Canada). Comparative analysis of the DNA sequence revealed complete sequence identity (100%) with reported *mecB* genes located either on plasmid pMCCL2 (*M. caseolyticus* JCSC5402; GenBank accession no. NC_011996.1) or within the SCC*mec*-like element of *M. caseolyticus* JCSC7096 (accession no. AB498756.1) (*15*,*16*). Apart from other *mecB* database entries, the highest nucleotide identity was shared with the sequence of *mecD* (68.7%), whereas the reported allotypes of *mecC* and *mecA* were more distantly related (Technical Appendix Figure 1). WGS revealed that the UKM4229 genome consists of a 2,851,374-bp circular chromosome and 2 different plasmids, a 20,725-bp plasmid (pSAWWU4229_2) and an 84,599-bp plasmid (pSAWWU4229_1; Figure); the latter carried *mecB* (GenBank accession no. PRJEB19527). The pSAWWU4229_1 plasmid backbone showed the highest similarity with the plasmid pMCLL2 of *M. caseolyticus* JCSC5402 (GenBank accession no. AP009486.1; blastn [https://blast.ncbi.nlm.nih.gov/Blast.cgi] 2.7.0+ maximum score 27,835; query coverage 71%; identity 99%) (*33*). These 2 plasmids shared 73.3% nucleotide identity (global alignment using Stretcher [Emboss], Matrix EDNAFULL; gap penalty 16, extend penalty 4). Whole plasmid comparative analysis of the sequences of pSAWWU4229_1 and pMCCL2 showed homologous regions between the *mec* gene complex, the downstream part of the *mec* complex, and the other antibiotic drug resistance genes (Technical Appendix Figure 2).

**Figure:**
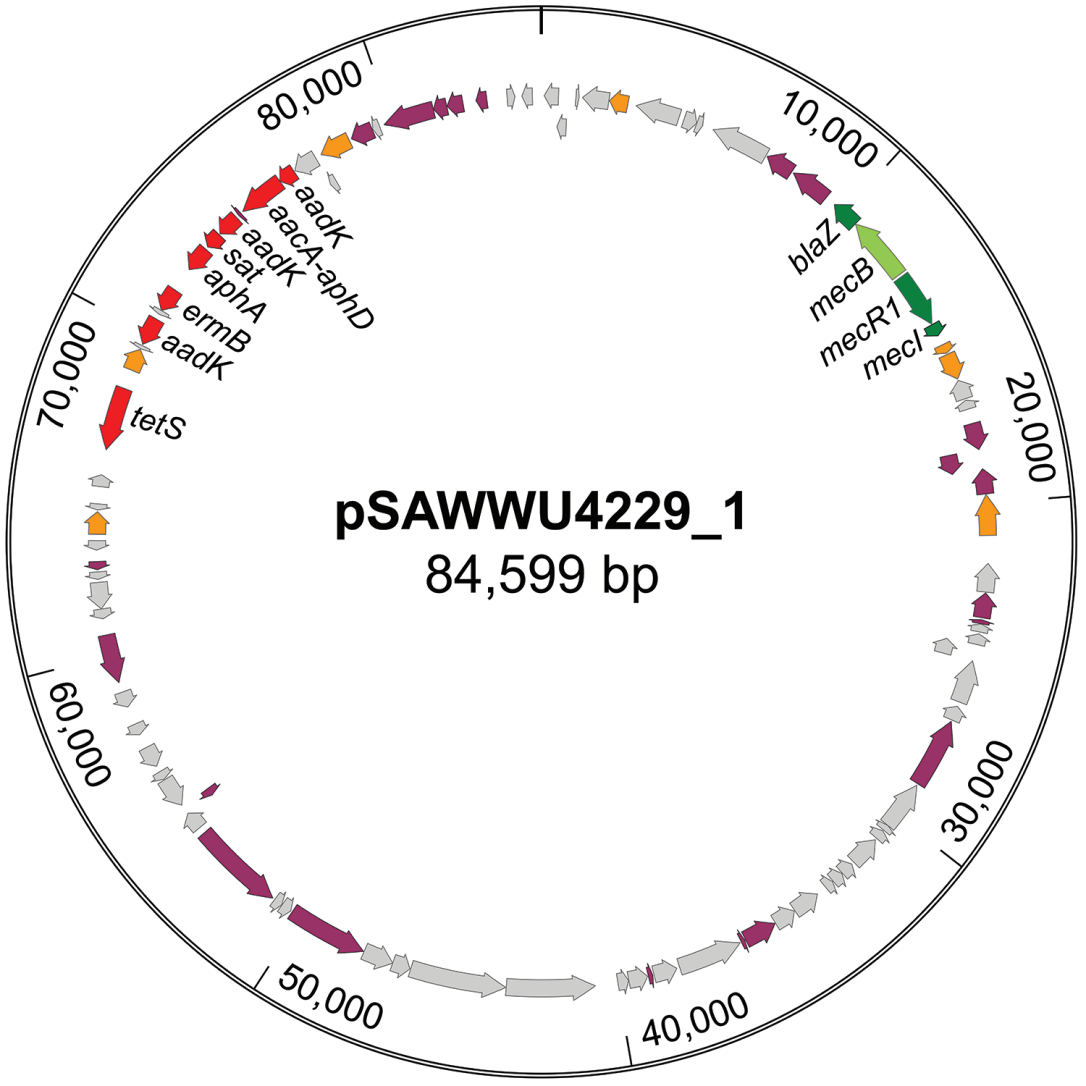
Circular map of the *mecB*-carrying plasmid pSAWWU4229_1 from *Staphylococcus aureus* isolate UKM4229, obtained from a 67-year-old cardiology inpatient who had no signs of infection, Münster, Germany. Arrows indicate annotated genes: the *mec*-complex is noted in green, antibiotic resistance genes in red, transposase/integrase genes in orange, other genes with known function in violet, and other genes with unknown function in gray.

Within the pSAWWU4229_1 plasmid, *mecB* was flanked by β-lactam regulatory genes similar to *mecR, mecI*, and *blaZ* (nucleotide identities: 99.9%, *mecRm* from *M. caseolyticus* JCSC7096; 100%, *mecIm* from *M. caseolyticus* JCSC7096 and 100%, *blaZm* from *M. caseolyticus* JCSC7096). pSAWWU4229_1 contained additional antibiotic drug resistance genes encoding resistance to aminoglycosides (*aacA-aphD*, *aphA*, and *aadK*), as well as macrolides (*ermB*), tetracyclines (*tetS*), and streptothricin (*sat*), all located in the same gene section. This particular region of the plasmid showed similarities with the transposon Tn551 of *S. aureus* 4578 (Genbank accession no. LC125350.1; blastn 2.7.0+ maximum score 11,064; query coverage 10%; identity 99%) (*34*). The sequences shared 48.9% nucleotide identity (global alignment using Stretcher [Emboss], Matrix EDNAFULL; gap penalty 16, extend penalty 4). Mating-pore genes or genes responsible for the DNA transfer suggesting self-transmission or mobilization properties of pSAWWU4229_1 were not detected. Genotyping revealed that *S. aureus* isolate UKM4229 belonged to multilocus sequence typing type ST7 and *spa*-type t091 (*spa*-CC 091).

DNA microarray analysis and WGS revealed the isolate possessed the leucotoxin genes *lukF, lukS*, *lukD*, *lukE*, *lukX*, and *lukY*. The isolate belonged to capsule type 8, and the biofilm-associated genes *icaA, C,* and *D* were detected. Furthermore, the *hlb*-converting bacteriophage of immune-evasion cluster type G comprising the enterotoxin encoding genes *sep*, *sak*, and *scn* was present in the genome. Additional information about the virulence profile of this isolate is given in Technical Appendix Table 2.

## Discussion

Recent studies have shown that mobile SCC*mec* elements have been imported more frequently by different *S. aureus* clonal lineages than previously assessed (*35*). Nevertheless, in contrast to the huge diversity of non-MRSA *S. aureus* clonal lineages (*36*), comparatively few clonal lineages still dominate the global MRSA population (*37*). However, an increased transferability of methicillin resistance by a plasmid-encoded course of action would have the capacity to drastically change the MRSA epidemiology. In staphylococci and other members of the phylum *Firmicutes*, plasmids have contributed enormously to the emergence and spread of antimicrobial resistance, and plasmid-encoded penicillin resistance has reached or exceeded 80% of clinical staphylococcal isolates (*38*).

In *M. caseolyticus*, *mecB* genes have been found within the chromosome as part of an SCC*mec* element as well as on a plasmid (*15*,*16*,*37*). For *S. aureus* UKM4229, it was shown that the *mecB* carrying plasmid pSAWWU4229_1 was distantly related to a macrococcal plasmid (pMCLL2 of *M. caseolyticus* JCSC5402), substantiating a possible gene transfer between the two genera. Because macrococcal and staphylococcal species may share the same hosts, mammalian skin and food, an exchange of mobile genetic elements between members of both closely related genera is likely and transmission to mammal-adapted staphylococci is generally to be feared (*3*). Genotyping of *S. aureus* UKM4229 revealed *spa*-type t091, which is relatively common, as 0.92% of the >370,000 submitted *spa* sequences assigned to ≈17,000 *spa* types (as of February 2017) of the RIDOM SpaServer database (http://spa.ridom.de/spatypes.shtml) belong to this *spa* type.

Routine phenotypic methods for susceptibility testing cannot distinguish between methicillin resistance determinants; thus, *mecB*-encoded methicillin resistance can remain undiscovered. Moreover, *mecB* detection is not part of molecular screening approaches. Certain clonal lineages of *S. aureus*, including MRSA, have emerged as zoonotic pathogens colonizing farm and wild animals (*40*). Tetracycline resistance frequently observed in staphylococci associated with husbandry is another indication for a possible livestock origin of the isolate (*41*). A putative livestock source of the *mecB*-encoding plasmid underlines the importance of the One Health concept in combating the spread of antimicrobial drug resistance.

Although the *mecB* isolate has been tested susceptible toward several agents of non–β- lactam antibiotic drug classes, the generally increased risk, compared to that of a SCC*mec* transfer, should be taken into consideration in that a *mecB*-encoding plasmid will be transmitted through horizontal gene transfer to other staphylococcal strains, even to already multidrug-resistant strains. In *S. aureus*, 2 major means of horizontal gene transfer for plasmids have been described: conjugation and bacteriophage transduction. Here, pSAWWU4229_1 did not harbor the typical genes responsible for conjugation or mobilization, which is, however, a common lack in *S. aureus*, affecting ≈95% of plasmids (*41*2 In contrast, for most staphylococcal plasmids, a transfer through bacteriophage generalized transduction has been suggested (*43*,*44*). Further studies are warranted to underpin this putative threat and to investigate how a plasmidborne methicillin resistance would affect the SCC*mec*-based methicillin resistance. For UKM4229, the WGS data revealed that the SCC*mec* chromosomal attachment site (*attB*) locus and the neighboring *orfX* (*rlmH*) gene were intact, and no integration of an SCC*mec* element was found.

The *mecB* isolate was tested to be susceptible to ceftobiprole and ceftaroline. Although cephalosporins with anti-MRSA activity are still active against the majority of MRSA isolates, nonsusceptibility has been already associated with certain MRSA lineages ranging between 3.9% and 33.5% of all MRSA isolates (*45*–*48*).

The discovery of plasmid-encoded methicillin resistance in *S. aureus* of probably macrococcal origin in a healthcare setting reveals a novel level of risk of the transfer of broad β-lactam resistance in staphylococci. Further studies are needed to clarify the real prevalence of *mecB*-caused methicillin resistance among MRSA and methicillin-resistant coagulase-negative staphylococci in human and animal populations, whether *mecA* and *mecC* genes could be found integrated on plasmids, and how the answers to these questions may affect human and animal health.

Technical Appendix Resistance and virulence profiles of *Staphylococcus aureus* UKM4229; phylogenetic relationships of *mec* genes conferring methicillin resistance and overview of characteristic features, and structural comparison of pSAWWU4229_1 and pMCCL2.
