# Supplementary material for: Plasmid-Encoded Transferable mecB-Mediated Methicillin Resistance in Staphylococcus aureus
Source: Emerg Infect Dis. 2018 Feb;24(2):242–8. doi: 10.3201/eid2402.171074 (PMC5782906; doi:10.3201/eid2402.171074)
Supplement: Technical Appendix — Resistance and virulence profiles of Staphylococcus aureus UKM4229; phylogenetic relationships of mec genes conferring methicillin resistance and overview of characteristic features, and structural comparison of pSAWWU4229_1 and pMCCL2. [file 17-1074-Techapp-s1.pdf]

Article DOI: https://doi.org/10.3201/eid2402.171074

Plasmid-Encoded Transferable *mecB*-Mediated Methicillin Resistance in *Staphylococcus aureus*

Technical Appendix

Technical Appendix Table 1. Resistance profile of *S. aureus* UKM4229 determined by microarray and whole genome sequencing*

		Result by			
			WGS		
Gene	Description†	Microarray	UKM4229 genome	pSAWWU4229_1	pSAWWU4229_2
Resistance genotype: methicillin					
mecA	Alternate penicillin binding protein 2, defining MRSA	–	–	–	–
mecB	Beta-lactam-inducible penicillin-binding protein	ND†	–	+	–
mecC	Novel *mecA* homologue, also associated with beta-lactam resistance	–	–	–	–
mecD	Beta-lactam-inducible penicillin-binding protein	ND	–	–	–
Resistance genotype: penicillinase					
blaZ	Beta-lactamase gene	+	–	+	+
blaZ-SCCmecXI	Beta-lactamase gene associated with SCCmec XI elements	–	–	–	–
blaI	Beta lactamase repressor (inhibitor)	+	–	–	+
blaR	Beta-lactamase regulatory protein	+	–	–	+
Resistance genotype: MLS antibiotics					
erm(A)	rRNA methyltransferase associated with macrolide/lincosamide resistance	–	–	–	–
erm(B)	rRNA methyltransferase associated with macrolide/lincosamide resistance	+	–	+	–
erm(C)	rRNA methyltransferase associated with macrolide/lincosamide resistance	–	–	–	–
lnu(A)	Lincosaminide nucleotidyltransferase (=*linA*)	–	–	–	–
msr(A)	Macrolide efflux pump	–	–	–	–
mef(A)	Macrolide efflux protein A	–	–	–	–
mph(C)	Macrolide phosphotransferase II (=*mpbBM*)	–	–	–	–
vat(A)	Virginiamycin A acetyltransferase	–	–	–	–
vat(B)	Acetyltransferase inactivating streptogramin A	–	–	–	–
vga(A)	ABC transporter conferring resistance to streptogramin A and related compounds	–	–	–	–
vga(A) (BM 3327)	*vga(A)* allele from strain BM 3327	–	–	–	–
vgB(A)	Virginiamycin B hydrolase (=*vgb*)	–	–	–	–
Resistance genotype: aminoglycosides					
aacA-aphD	Aminoglycoside adenyl-/phosphotransferase (gentamicin, tobramycin)	+	–	+	–
aadD	Aminoglycoside adenyltransferase (neo-/ kanamycin, tobramycin)	–	–	–	–
aphA3	Aminoglycoside phosphotransferase (neo-/ kanamycin)	+	–	+	–
Resistance genotype: miscellaneous genes					
sat	Streptothricin acetyltransferase	+	–	+	–
dfrS1	Dihydrofolate reductase mediating trimethoprim resistance (=*dfrA*)	–	–	–	–
fusB	Fusidic acid resistance gene (= *far1*)	–	–	–	–
fusC	Fusidic acid resistance gene (= Q6GD50)	–	–	–	–
mupA	Isoleucyl-tRNA synthetase associated with mupirocin resistance (=*mupR*)	–	–	–	–
tet(K)	Tetracycline efflux protein	–	–	–	–
tet(M)	Ribosomal protection protein associated with tetracycline resistance	–	–	–	–
tetS	Tetracycline resistance protein TetS	ND	–	+	–
cat (total)	Chloramphenicol acetyltransferase	–	–	–	–
cfr	23S rRNA methyltransferase (phenicols, lincosamides, oxazolidinones, pleuromutilins, streptogramin A)	–	–	–	–
fexA	Chloramphenicol/florfenicol exporter	–	–	–	–
fosB	Metallothiol transferase	–	–	–	–

Gene	Description†	Result by Microarray	Result by WGS: UKM4229 genome	Result by WGS: pSAWWU4229_1	Result by WGS: pSAWWU4229_2
fosB (plasmid)		–	–	–	–
Resistance genotype: efflux systems					
qacA	Quaternary ammonium compound/multidrug efflux protein A	–	–	–	–
qacC (total)	Quaternary ammonium compound/multidrug efflux protein C	–	–	–	–
sdrM	Multidrug efflux pump	+	+	–	–
Resistance genotype: glycopeptides					
vanA	Vancomycin resistance gene	–	–	–	–
vanB	Vancomycin resistance gene from enterococci and *Clostridium*	–	–	–	–
vanZ	Teicoplanin resistance gene from enterococci	–	–	–	–

*+, positive; –, negative; ND, not determined; WGS, whole genome sequencing
†Description as provided by the manufacturer (*S. aureus* Genotyping Kit 2.0 manual, Alere Technologies GmbH, Jena, Germany), except for ND cases

Technical Appendix Table 2. Virulence profile of *S. aureus* UKM4229 determined by microarray and whole genome sequencing*

Gene	Description†	Result by Microarray	Result by WGS: UKM4229 genome	Result by WGS: pSAWWU4229_1	Result by WGS: pSAWWU4229_2
Virulence: enterotoxins					
sea	Enterotoxin A (= *entA*)	–	–	–	–
sea (320E)	Enterotoxin A, allele from strain 320E	–	–	–	–
seb	Enterotoxin B (= *entB*)	–	–	–	–
sec	Enterotoxin C (= *entC*)	–	–	–	–
sed	Enterotoxin D (= *entD*)	–	–	–	–
see	Enterotoxin E (= *entE*)	–	–	–	–
seg	Enterotoxin G (= *entG*)	–	–	–	–
seh	Enterotoxin H (= *entH*)	–	–	–	–
sei	Enterotoxin I (= *entI*)	–	–	–	–
sej /*selj*	Enterotoxin J (= *entJ*)	–	–	–	–
sek / *selk*	Enterotoxin K (= *entK*)	–	–	–	–
sel / *sell*	Enterotoxin L (= *entL*)	–	–	–	–
selm	Enterotoxin-like gene/protein M (= *sem*, *entM*)	–	–	–	–
seln (consensus)	Enterotoxin-like gene/protein N (= *sen*, *entN*), consensus probe	–	–	–	–
selo	Enterotoxin-like gene/protein O (= *seo*, *entO*)	–	–	–	–
selp / *sea* (N315)	Enterotoxin A, allele from strain N315 (= enterotoxin P, *entP*)	+	+	–	–
egc	Enterotoxin gene cluster, consisting of *seg*, *sei*, *selm*, *seln*, *selo*, *selu*	–	–	–	–
seq / *selq*	Enterotoxin Q (= *entQ*)	–	–	–	–
ser	Enterotoxin R (= *entR*)	–	–	–	–
selu	Enterotoxin-like gene/protein U (= *seu*, *entU*)	–	–	–	–
Virulence: hemolysin gamma and leukocidins					
lukF / *hlgB*	Hemolysin gamma / leukocidin, component B (F)	+	+	–	–
lukS / *hlgC*	Hemolysin gamma / leukocidin, component C (S)	+	+	–	–
lukS (ST22+ST45)	Hemolysin gamma / leukocidin, component C (S), allele from ST22 and ST45	+	+	–	–
hlgA	Hemolysin gamma, component A	+	+	–	–
lukF-PV	Panton Valentine leukocidin F component	–	–	–	–
lukS-PV	Panton Valentine leukocidin S component	–	–	–	–
lukF-PV (P83)	F component of leukocidin from ruminants	–	–	–	–
lukM	S component of leukocidin from ruminants	–	–	–	–
lukD	Leukocidin D component	+	+	–	–
lukE	Leukocidin E component	+	+	–	–
lukX	Leukocidin/hemolysin toxin family protein (= *lukA* or *lukG*)	+	+	–	–
lukY	Leukocidin/hemolysin toxin family protein (= *lukB* or *lukH*)	+	+	–	–
lukY (ST30+ST45)	Leukocidin/hemolysin toxin family protein (= *lukB* or *lukH*),	–	–	–	–

Gene	Description†	Result by: Microarray	Result by: WGS: UKM4229 genome	Result by: WGS: pSAWWU4229_1	Result by: WGS: pSAWWU4229_2
	allele from ST30 and ST45				
	Virulence: hemolysins				
hla / *hly*	Hemolysin alpha	+	+	–	–
hlb	Hemolysin beta	+	+	–	–
undisrupted *hlb*	Hemolysin beta without phage insertion	–	–	–	–
hld	Hemolysin delta	ND	+	–	–
	Virulence: hlb-converting phage				
sak	Staphylokinase	+	+	–	–
chp	Chemotaxis-inhibiting protein (CHIPS)	–	–	–	–
scn	Staphylococcal complement inhibitor	+	+	–	–
	Virulence: exfoliative toxins				
etA	Exfoliative toxin serotype A	–	–	–	–
etB	Exfoliative toxin serotype B	–	–	–	–
etD	Exfoliative toxin D	–	–	–	–
	Virulence: epidermal cell differention inhibitors				
edinA	Epidermal cell differentiation inhibitor	–	–	–	–
edinB	Epidermal cell differentiation inhibitor B	–	–	–	–
edinC	Epidermal cell differentiation inhibitor C	–	–	–	–
	Virulence: ACME locus				
ACME cluster	Arginine catabolic mobile element	–	–	–	–
arcA-SCC	ACME-locus: arginine deiminase	–	–	–	–
arcB-SCC	ACME-locus: ornithincarbamoyltransferase	–	–	–	–
arcC-SCC	ACME-locus: carbamatkinase	–	–	–	–
arcD-SCC	ACME-locus: arginine/ornithine-antiporter	–	–	–	–
	Virulence: proteases				
aur (consensus)	Aureolysin	+	+	–	–
aur (other than MRSA252)		+	+	–	–
aur (MRSA252)		–	–	–	–
splA	Serinprotease A	+	+	–	–
splB	Serinprotease B	+	+	–	–
splC	Serinprotease C	ND	+	–	–
splE	Serinprotease E	+	+	–	–
splF	Serinprotease F	ND	+	–	–
sspA	Glutamylendopeptidase	+	+	–	–
sspB	Staphopain B, protease	+	+	–	–
sspP (consensus)	Staphopain A (staphylopain A), protease	+	+	–	–
	Capsule- and biofilm-associated genes				
cap 1 (total)	Capsule type 1	–	–	–	–
cap 5 (total)	Capsule type 5	–	–	–	–
cap 8 (total)	Capsule type 8	+	+	–	–
capH8	Capsular polysaccharide synthesis enzyme	+	+	–	–
capI8	Capsular polysaccharide biosynthesis protein	+	+	–	–
capJ8	O-antigen polymerase	+	+	–	–
capK8	Capsular polysaccharide biosynthesis protein	+	+	–	–
icaA	Intercellular adhesion protein A	+	+	–	–
icaC	Intercellular adhesion protein C	+	+	–	–
icaD	Biofilm PIA synthesis protein D	+	+	–	–
bap	Surface protein involved in biofilm formation	–	–	–	–
	Adhesion factors/MSCRAMM genes				
bbp (total)	Bone sialoprotein-binding protein	+	+	–	–
clfA (total)	Clumping factor A	+	+	–	–
clfB (total)	Clumping factor B	+	+	–	–
cna	Collagen-binding adhesin	–	–	–	–
ebh (consensus)	Cell wall associated fibronectin-binding protein	+	+	–	–
ebpS (total)	Cell surface elastin binding protein	+	+	–	–
eno	Enolase	+	+	–	–
fib	Fibrinogen binding protein (19 kDa)	+	+	–	–
fnbA (total)	Fibronectin-binding protein A	+	+	–	–

		Result by			
			WGS		
Gene	Description†	Microarray	UKM4229 genome	pSAWWU4229_1	pSAWWU4229_2
fnbB (total)	Fibronectin-binding protein B	+	+	–	–
map (total)	Major histocompatibility complex class II analog protein (= Extracellular adherence protein, *eap*)	+	+	–	–
sasG (total)	*Staphylococcus aureus* surface protein G	–	–	–	–
sdrC (total)	Ser-Asp rich fibrinogen-/bone sialoprotein-binding protein C	+	+	–	–
sdrD (total)	Ser-Asp rich fibrinogen-/bone sialoprotein-binding protein D	+	+	–	–
sdrE	Ser-Asp rich fibrinogen-/bone sialoprotein-binding protein E	ND	+	–	–
sdrF	Ser-Asp rich fibrinogen-/bone sialoprotein-binding protein F	ND	–	+	–
vwb (total)	Van Willebrand factor binding protein	+	+	–	–

*+, positive; –, negative; ND, not determined; MSCRAMM, microbial surface components recognizing adhesive matrix molecules; WGS, whole genome sequencing.

†Description as provided by the manufacturer (*S. aureus* Genotyping Kit 2.0 manual, Alere Technologies GmbH, Jena, Germany), except for ND cases

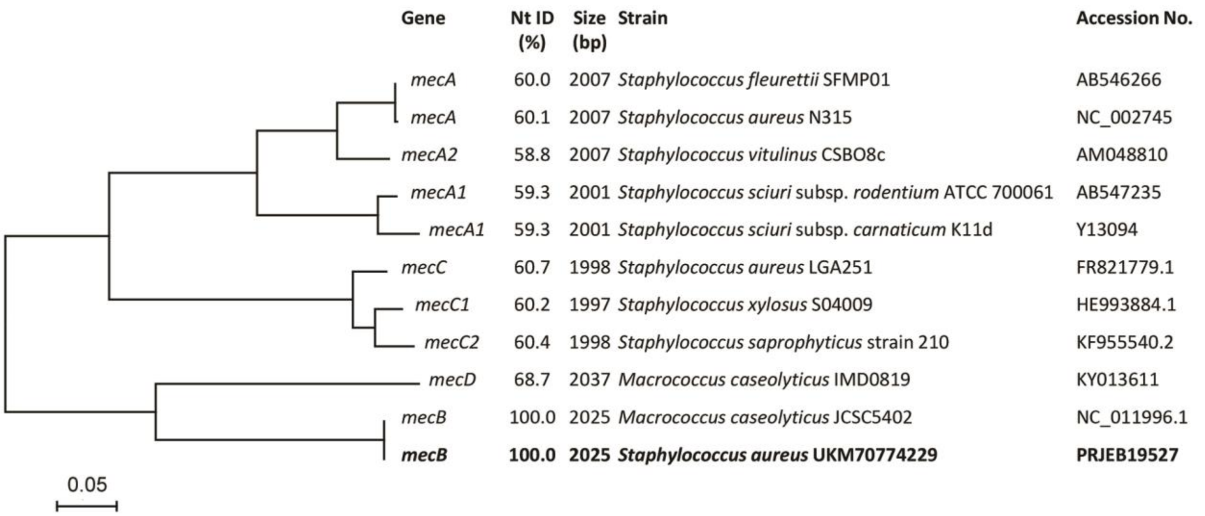

Technical Appendix Figure 1. Phylogenetic relationships of *mec* genes conferring methicillin resistance and overview of characteristic features. Evolutionary history was inferred by using the maximum likelihood method based on the Tamura-Nei model (*1*) in MEGA7 (*2*). Nucleotide sequences were aligned using MUSCLE (*3*). The tree is drawn to scale, with branch lengths measured in the number of substitutions per site. Nucleotide identity (nt ID) between the *mecB* gene in strain UKM4229 and other *mec* genes was determined by sequence alignment using Clustal OMEGA (http://www.ebi.ac.uk/Tools/msa/clustalo/).

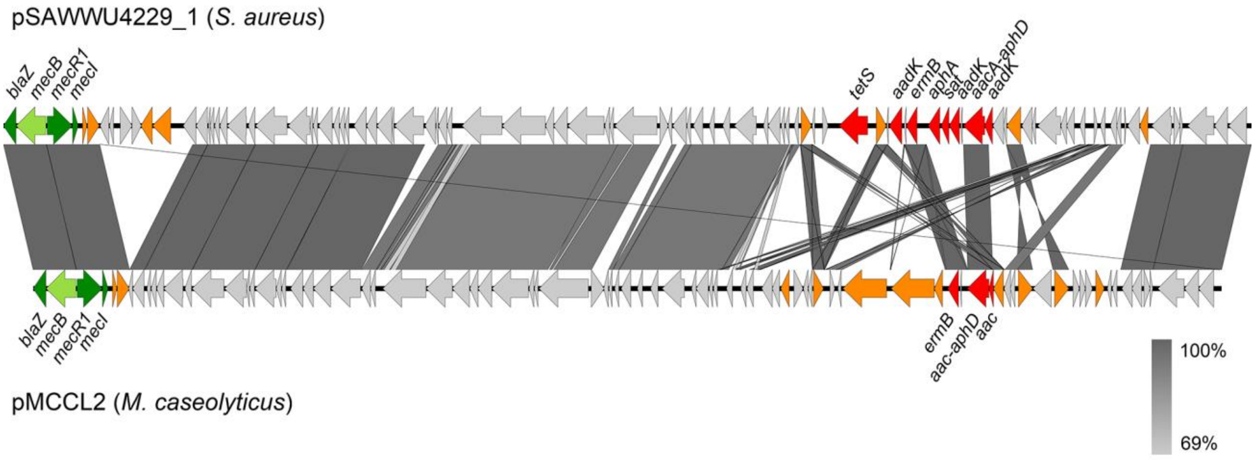

Technical Appendix Figure 2. Structural comparison of pSAWWU4229_1 and pMCCL2 (*M. caseolyticus* JCSC5402; NC_011996.1) performed by Easyfig software (*49*). Gray areas represent regions with nucleotide sequence similarities ranging between 69% and 100%. The *mec*-complex is colored in green, antibiotic resistance genes in red, and transposase/integrase genes in orange.

References

<jrn>1. Tamura K, Nei M. Estimation of the number of nucleotide substitutions in the control region of mitochondrial DNA in humans and chimpanzees. Mol Biol Evol. 1993;10:512–26.</jrn>

<jrn>2. Kumar S, Stecher G, Tamura K. MEGA7: Molecular Evolutionary Genetics Analysis Version 7.0 for bigger datasets. Mol Biol Evol. 2016;33:1870–4. http://dx.doi.org/10.1093/molbev/msw054</jrn>

<jrn>3. Edgar RC. MUSCLE: multiple sequence alignment with high accuracy and high throughput. Nucleic Acids Res. 2004;32:1792–7. http://dx.doi.org/10.1093/nar/gkh340</jrn>
